# Regional Anesthesia in Circumcision Surgery: Which of the Two Things Is Better?

**DOI:** 10.5152/eurasianjmed.2022.20402

**Published:** 2022-02-01

**Authors:** Mehmet Selim Çömez, Aydın Pelin

**Affiliations:** 1 Department of Anesthesiology and Reanimation, Hatay Mustafa Kemal University Tayfur Ata Sökmen School of Medicine, Hatay, Turkey; 2 Department of Anesthesiology and Reanimation, Health Sciences University, Region Training and Research Hospital, Erzurum, Turkey

**Keywords:** Caudal block, Penil block, circumcision, FLACC pain scale

## Abstract

**Objective:** Postcircumcision pain in children can cause restlessness, crying and bleeding due to trauma. However, there are various methods to prevent postoperative pain, caudal and penile blocks are in the foreground. The primary objective of this study is to evaluate the effectiveness of CB and PB for the relief of postcircumcision pain. The secondary aim is to evaluate the postoperative additional analgesic requirement and side effects of these blocks.

**Materials and Methods:** A total of 148 children between the ages of 2 and 10 who underwent circumcision surgery were randomly assigned to two groups in terms of postoperative analgesia. 1) A group of caudal block (0,5 ml/kg %0.25 levobupivacaine) and 2) A group of penile block (0,3 ml/kg %0,25 levobupivacaine). Premedication and sedoanalgesia were standardized. The pain (FLACC Pain Score), analgesic consumption, motor block (Bromage Scale) and side effects (vomiting, hematoma, urinary retention) were assessed postoperatively for 4 hours.

**Results:** Postoperative FLACC scores were lower for caudale block group in the 1st, 3rd and 4th hours. There was no significant difference in postoperative analgesic consumption between the groups. The most common postoperative side effect was vomiting in both groups.

**Conclusion:** Caudal block provided more effective analgesia than penile block in postcircumcision pain control.

**Main Points**

- Although both caudal block (CB) and penile block (PB) provided sufficient and effective analgesia, CB provided better analgesia.
- Both methods had a low complication rate.
- Since the frenulum is innervated by the perineal branch of the pudendal nerve as well as the penile nerve, adequate analgesia may not be achieved only with the PB.

## Introduction

Circumcision is the surgical removal of the foreskin in the distal penis. It is one of the most common outpatient surgery in the world since it is related to the prevention of urinary tract infections, acquisition of human immunodeficiency virus, sexually transmitted infections, penile cancer, and religious rituals.^1^ The aim of ideal anesthesia is to provide motor block and analgesia with minimal damage to physiology and metabolism, as well as to ensure rapid recovery and early return to normal life. Especially in the postoperative early period, postcircumcision pain leads to crying, agitation, bleeding due to trauma, and it triggers physiological stress response.^2^ The most common methods used in postcircumcision pain are caudal block (CB), penile block (PB), topical analgesia, and administration of drugs such as opioids, Nonsteroidal anti-inflammatory drug (NSAIDs) etc.^3,4^ Long-acting opioids are not recommended for children who undergo outpatient surgery because of developed side effects from nausea and vomiting to breathing depression.^5^

Caudal block and penile block diminish the somatic pain, so physiologic stress response and opioid consumption are reduced. Mobilization is achieved in the early postoperative period.^6^ Caudal block is one of the most popular postoperative analgesic techniques on postoperative pain after penile surgeries besides lower abdominal and scrotal surgeries in the childhood period.^7^ Penile block is also often preferred in circumcision, minor hypospadias, and other penile procedures, but it is not still clear which one is superior.

The primary objective of this study is to evaluate the effectiveness of CB and PB for the relief of postcircumcision pain. The secondary aim is to evaluate the postoperative additional analgesic requirement and side effects of these blocks.

## Materials and Methods

After the approval of the local clinical research ethics committee (date: December 1, 2015, and issue: 37732058-53/7017), this prospective, randomized double-blind study was conducted at the pediatric surgery clinic and operating theater in our hospital between December 2015 and February 2016. Informed consent form was received from parents after a detailed explanation of the procedures to be performed on children.

A total of 158 children who were American Society of Anesthesiologists Physical Status Score (ASA) I-II and between 2 and 10 years were scheduled to be circumcised in this study. Children were excluded if they had infection in the intervention area, severe systemic disease, pre-existing neurological and spinal diseases, bleeding diathesis, a history of seizure disorder, or known hypersensitivity to local anesthetics (LAs). Randomization was applied by closed envelope method, and the cases were divided into 2 groups as caudal group (group C, n = 74) and penile group (group P, n = 74).

Premedication was performed with 0.5 mg/kg peroral midazolam 45 minutes before the operation. Children were monitored with electrocardiogram (ECG), non-invasive blood pressure (NIBP), heart rate (HR), oxygen saturation, and a 22-G intravenous catheter was inserted into a peripheral vein in the operation room. Electrolyte solution with 0.45-5% dextrose (isolen P 500 mL, Polypharma, Istanbul, Turkey) (3-5 mL/kg/h) was given intravenously. Sedoanalgesia was given to all cases by 0.1 mg/kg midazolam (demizolam 1 mg/mL, DEM Medical, Istanbul, Turkey) and 2 mg/kg ketamine (ketalar 50 mg/mL, Pfizer, Istanbul, Turkey) intravenously, and 4 L/min O_2_ was given with the face mask. The blocks were made 10 minutes before the surgery. Patients in group P were placed in the supine position and the pubic region was sterilized. A 25-G, 30-mm-long needle was inserted into the lateral edge of the symphysis pubis, just below both pubic ramus, 10-15˚ medial to the vertical axis and caudal direction. A “pop” was felt as it passed the scarpa fascia approximately 8-30 mm under the skin. The prepared solution (0.25% levobupivacaine, 0.15 mL/kg) was injected into each side separately.^8^ After the sacral area was sterilized, the patients in group C were placed in lateral decubitus position with the neck flexed, and the knees were drawn up to the abdomen. Coccyx was palpated. Sacral hiatus was felt depressed between the sacral cornua. The needle (18 G Tuohy, Caufix, Egemen, Izmir, Turkey) was advanced approximately 5 mm at an angle of 20-30˚ into the sacral hiatus. A “pop” was felt as the sacrococcygeal membrane was passed through. The stylet of the Tuohy needle was removed. It was confirmed by aspiration that cerebrospinal fluid did not come. The prepared solution (0.25% levobupivacaine, 0.5 mL/kg) was injected.^9^ The absence of subcutaneous bulging and resistance to injection was observed. The block was checked with the pinprick test. Also, cases with 20% increase in HR were considered as unsuccessful blocks and these patients were removed from the study.

Vital findings (ECG, NIBP, HR, SPO_2_) of cases were recorded at 5-minute intervals during the operation. Face, Legs, Activity, Cry, Consolability (FLACC) Behavioural pain scale, analgesic consumption, and side effects such as nausea, vomiting, hematoma, and urinary retention were evaluated and recorded postoperatively for 4 hours in the outpatient ward by a different researcher, who had no knowledge of the groups, at 1-hour intervals, and side effects were queried from parents of the patients after 24 hours. Paracetamol (parol 10 mg/mL flacon, Atabay, Istanbul, Turkey) of 15 mg/kg was intravenously given to the cases with FLACC pain score of 4 and above as rescue analgesia. The cases were discharged postoperatively 4-6 hours without any problems.

Considering the medium effect size (0.50) as a general approach in the pre-study evaluation, the number of patients required to be included in each group for 80% power was found to be 64. However, in case of data loss, the study was carried out with 74 patients. Statistical analysis was performed using IBM Statistical Package for the Social Sciences Statistics v.22.0 (IBM SPSS Corp.; Armonk, NY, USA) software package. The normality distribution of variables was checked with the Kolmogorov–Smirnov and histogram tests. Descriptive data were expressed as mean ± standard deviation. Categorical variables were analyzed using the chi-square test. Normally distributed data comprising continuous variables were analyzed using the Student’s *t*-test. For the statistical analysis, *P* < .05 was considered statistically significant.

## Results

A total of 158 children between 2 and 10 years were scheduled to be circumcised in this study. The parents of 2 children refused to participate in the study. Three children were not included in the study due to upper airway infection. One child was also excluded from the study because of epilepsy. In addition, 4 cases were excluded from the study by changing the anesthesia method due to unsuccessful block (in group P), accompanying inguinal hernia and orchiopexy (laryngeal mask airway (LMA) and 0.75 mL/kg fluid for CB) (Figure 1). A total of 148 patients were included who met the criteria for the study. No significant differences existed between the groups with respect to age, weight, and duration of surgery (Table 1).

Postoperative FLACC scores in the first, third, and fourth hours in group C were significantly lower than group P (*P* < .05) (Table 2). Postoperative FLACC scores in the second hour in group C were lower than group P, but there was no significant difference statistically (*P* = .054) (Table 2). No hematoma, motor block, and urinary retention were seen in either group. In total, 6 children vomited in group C and 8 in group P. There were no statistically significant differences between the groups for incidence of postoperative vomiting during the first 4 hours after the operation (*P* = .574) (Table 3). In group P, since FLACC score in 2 cases is 4, 15 mg/kg paracetamol was given intravenously. There was no need for additional analgesia in group C. There was no significant difference in the need for additional analgesia between the groups (*p* = .154) (Table 3). All of the patients were discharged on the same day after being comfortable, mobile, tolerating oral fluid, and passing urine.

## Discussion

Overtime, many methods have been used for anesthesia and analgesia in postcircumcision pain. Currently, regional techniques are in the foreground. Mainly used techniques are CB, PB, and penile ring infiltration.^5^ The superiority of other regional methods over penile ring infiltration is clearly stated.^10,11^ However, the superiority of CB and penile PB to each other cannot be clearly determined.

CB and PB are a routine application of our clinic for the relief of postoperative pain in circumcision cases. Although both methods demonstrated in this study provided effective analgesia, the CB provided more effective analgesia. There was no significant difference between the groups in terms of additional analgesia need and side effects. Consistent with our study, in a study conducted on circumcision cases, it was reported that postoperative Children’s Hospital of Eastern Ontario Pain Scale (CHEOPS) pain score was lower in CB than PB.^12^ Another study reported that CB performed in hypospadias cases provides higher success rate, better quality, and longer analgesia in postoperative pain control from PB.^13^ This is likely due to the fact that PB provides analgesia just on the half of glans, priapism, and penis by sensorial innervation of the penis from second, third, and fourth sacral roots.^14^ In addition, the CB spreads over a wider area because it targets the epidural space, thus providing effective analgesia for the entire region.^5^

In a retrospective study involving 738 patients covering the years 2013-2015, it was stated that CB provided better regional anesthesia in terms of narcotic analgesic need and FLACC score. In addition, it was reported that patients who took (dorsal penile nerve block (DPNB) used narcotic analgesia 4.2 times more.^15^ In our study, since the FLACC score was above 4 in 2 patients in the PB group, 15 mg/kg i.v. paracetamol was given. There was no need for additional analgesia in the CB group. However, there was no difference between the groups in terms of additional analgesic use (*P* = .154). A meta-analysis of 9 randomized, controlled trials involving 574 children aged 18 months to 16 years found similar analgesic success rates in CB and PB. In addition, CB was found to be associated with longer analgesia time but with prolonged urinary retention and walking delay.^16^ In our study, while CB provided more effective analgesia, motor block and urinary retention did not develop. This difference may be due to the meta-analysis being compiled from studies with different LAs, different doses, different concentrations, and different volumes. We preferred low concentration levobupivacaine because it provides a longer sensory block and shorter motor block.^17,18^

One study found no analgesic difference between CB and PB, although they used the same dose and concentration as levobupivacaine as we did.^19^ Another study reported that both methods achieved adequate analgesia.^17^ In a study comparing DPNB-US (DPNB by ultrasound) and CB, it was stated that DPNB-US was more effective. This was attributed to the fact that the LA dispersion was clearly visible in the tissue.^20^ Another study found that the efficacy and safety of DPNB-US with perineal approach and CB were similar. It was reported that perineal approach DPNB-US can be an alternative to CB in circumcision surgery.^21^ We did not use US in our study. However, we observed that CB was more effective than DPNB. One reason for this is that CB provides full penile analgesia, whereas PB provides analgesia in 3/4 of the dorsal penis.^22^ The other reason is the DPN, a branch of the pudendal nerve, that extends ventrolaterally from the glans to innervate the glans, including the frenulum, but the frenulum is also innervated by the perineal branch of the pudendal nerve.^23^ Therefore, the perineal branch of the pudendal nerve may not be blocked in DPNB.

Both regional techniques have potential complications. Caudal block can cause motor block, delayed first micturition, and an incidence of nausea and vomiting varying from 12% to 37%.^24^ Penile block can cause local hematoma, mild local edema, systemic toxic effects due to absorption of the LA,^25^ and rarely ischemia of the glands due to arterial compression,^26^ or the vasoconstrictor property of the LA.^27^ We did not encounter any technical difficulties, major complications, or neurological sequelae during CB or PB. The incidence of minor complications in our study was similar to these studies. In our study, motor block was not visible among CB group and hematoma of the penis in the PB group. In our study, 8 (10.8%) children vomited in group P and 6 (8.1%) in group C. However, there is no statistically significant difference between the groups (*P* = .574).

Almost all of such studies have been combined with general anesthesia. Although it is beyond the purpose of the study, one of the advantages of the studying method was that endotracheal tube (ETT) or LMA was not applied at all and a volatile agent was not given. Caudal block or PB with sedoanalgesia preserving spontaneous respiration ensured adequate and effective analgesia. Therefore, children are not exposed to the effects of volatile agents such as cognitive dysfunction, negative neuronal development and delayed compilation, and other effects such as croup and bronchospasm due to the use of ETT and LMA.

The most important limitation of this study was that the postoperative follow-up was only for 4 hours due to the fact that our patients underwent outpatient surgery. The other limitations were that our study was done only in male children and only between the ages of 2 and 10. We also did not evaluate different volumes and concentrations. Although the scoring systems where patients report their pain ratings are the gold standard, we used the preferred FLACC pain score. Since, this is impossible for children who are from 2 to 10 years and have included in our study.

In conclusion, this study supports the fact that these 2 methods provide adequate and effective analgesia after circumcision. Supplementary analgesic need is not observed in CB, and FLACC score in CB is lower than that in PB. Thus, we believe that the CB with levobupivacaine was better than PB in terms of postoperative circumcision analgesia in children.
